# Supplementary material for: Conditional chemoconnectomics (cCCTomics) as a strategy for efficient and conditional targeting of chemical transmission
Source: eLife. 2024 Apr 30;12:RP91927. doi: 10.7554/eLife.91927 (PMC11060718; doi:10.7554/eLife.91927)
Supplement: Figure 3—source data 3. [file elife-91927-fig3-data3.zip › Figure 3 source data3 part 1.zip!/Figure 3-Source data 3-part1/1175-9-CRISPResso_Report_rtnd62ko.zip!/CRISPResso_on_1175_12/1c.Alignment_barplot.pdf]

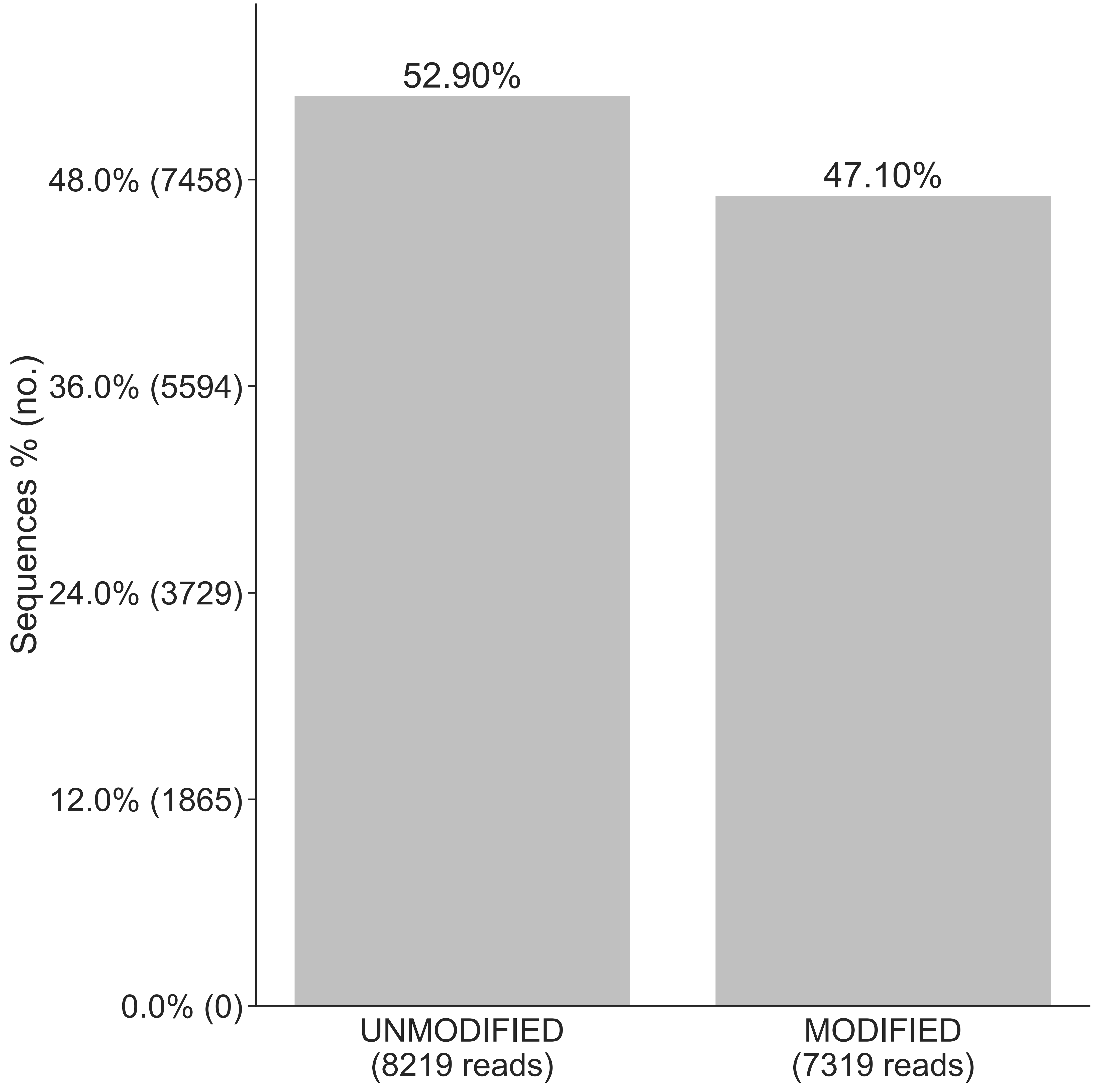

Sequences % (no.)
48.0% (7458)
36.0% (5594)
24.0% (3729)
12.0% (1865)
0.0% (0)
52.90%
47.10%
UNMODIFIED
(8219 reads)
MODIFIED
(7319 reads)
